# Supplementary material for: Porphyrin Functionalized Carbon Quantum Dots for Enhanced Electrochemiluminescence and Sensitive Detection of Cu2+
Source: Molecules. 2023 Feb 2;28(3):1459. doi: 10.3390/molecules28031459 (PMC9919192; doi:10.3390/molecules28031459)
Supplement: Supplementary file 1 [file molecules-28-01459-s001.zip › molecules-2186758-supplementary.pdf]

molecules

MDPI

Supporting Information

Porphyrin Functionalized Carbon Quantum Dots for Enhanced Electrochemiluminescence and Sensitive Detection of Cu^{2+}

Xinying Zhang [1,2], Xialing Hou [1,2], Decheng Lu [1,2], Yingying Chen [1,2] and Lingyan Feng [1,2,*]

[1] Materials Genome Institute, Shanghai University, Shanghai 200444, China
[2] Shanghai Engineering Research Center of Organ Repair, Shanghai 200444, China
[*] Correspondence: lingyanfeng@t.shu.edu.cn; Tel.: +86-21-66136189

And it was illustrated as follows: (M represents CQDs-TMPyP)

(1) Reduction reaction on electrode surface:

M + e- →CQDs•-

$S_2O_8^{2-}$ + e- →$SO_4^{\bullet -}$ + SO_4^{2-}.

(2) The formation of excited states:

M $^{\bullet -}$ + $SO_4^{\bullet -}$ →M* + SO_4^{2-}

(3) The excited state returns to the ground state:

M* →M + hv

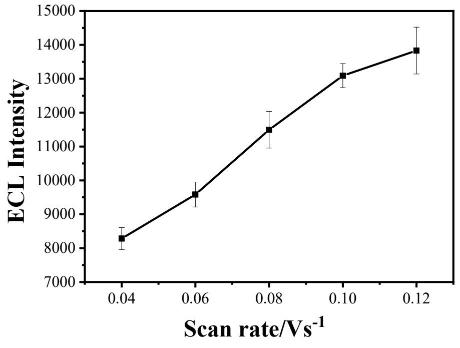

Figure S1. The ECL response of this biosensor for different scan rates.

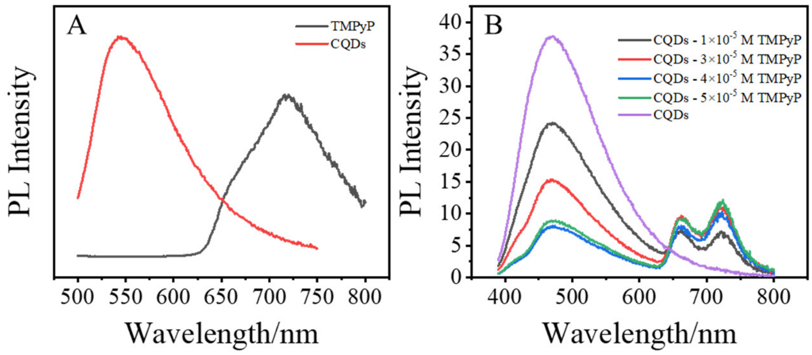

Figure S2. The fluorescence of CQDs and TMPyP; CQDs-TMPyP. (A) The fluorescence of CQDs (excitation peak 370 nm) and TMPyP (excitation peak 480 nm); (B) The fluorescence of CQDs-TMPyP with adding different amount of TMPyP (excitation peak 370 nm).

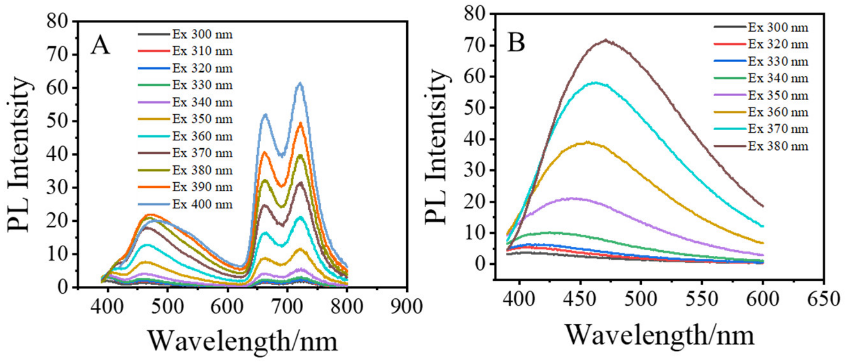

Figure S3. The florescence of TMPyP (A) and CQDs (B) at different excitation peaks.
